# Differentially-Expressed Genes Associated with Faster Growth of the Pacific Abalone, *Haliotis discus hannai*

**DOI:** 10.3390/ijms161126042

**Published:** 2015-11-18

**Authors:** Mi-Jin Choi, Gun-Do Kim, Jong-Myoung Kim, Han Kyu Lim

**Affiliations:** 1 Department of Fishery Biology, PuKyong National University, Busan 608-737, Korea; coawls@pukyong.ac.kr; 2 Department of Microbiology, PuKyong National University, Busan 608-737, Korea; gundokim@pknu.ac.kr; 3 Department of Marine and Fisheries Resources, Mokpo National University, Muan 534-729, Korea

**Keywords:** pacific abalone, growth, differentially expressed genes

## Abstract

The Pacific abalone *Haliotis discus hannai* is used for commercial aquaculture in Korea. We examined the transcriptome of Pacific abalone *Haliotis discus hannai* siblings using NGS technology to identify genes associated with high growth rates. Pacific abalones grown for 200 days post-fertilization were divided into small-, medium-, and large-size groups with mean weights of 0.26 ± 0.09 g, 1.43 ± 0.405 g, and 5.24 ± 1.09 g, respectively. RNA isolated from the soft tissues of each group was subjected to RNA sequencing. Approximately 1%–3% of the transcripts were differentially expressed in abalones, depending on the growth rate. RT-PCR was carried out on thirty four genes selected to confirm the relative differences in expression detected by RNA sequencing. Six differentially-expressed genes were identified as associated with faster growth of the Pacific abalone. These include five up-regulated genes (including one specific to females) encoding transcripts homologous to incilarin A, perlucin, transforming growth factor-beta-induced protein immunoglobulin-heavy chain 3 (ig-h3), vitelline envelope zona pellucida domain 4, and defensin, and one down-regulated gene encoding tomoregulin in large abalones. Most of the transcripts were expressed predominantly in the hepatopancreas. The genes identified in this study will lead to development of markers for identification of high-growth-rate abalones and female abalones.

## 1. Introduction

Abalone is regarded as a commercially-important marine gastropod with a high nutritional value [1]. Among the six abalone species (*Haliotis (H.) discus hannai*, *H. discus discus*, *H. madaka*, *H. gigantea*, *H. diversicolor supertexta*, and *H. diversicolor*) widely distributed in Korea, the Pacific abalone, *Haliotis discus hannai*, is the major species used in the aquaculture industry. Farmed abalone production in Korea has increased rapidly from 20 tons in 2000 to 9000 tons in 2012, and accounts for 20% of global abalone production [2,3]. However, the Korean abalone aquaculture industry has in recent years experienced a decline in productivity, possibly due to deterioration of aquaculture environments, including overcrowded facilities, and genetic degradation, such as inbreeding depression. Optimization of the culture conditions and increasing the nutritional value of abalone feed have been suggested as methods of improving productivity [4,5].

Selection of abalone species with higher growth rates is critical to reduce the time and cost involved in reaching a marketable size in the aquaculture industry. Much effort has focused on developing a fast-growing strain by selective breeding [6] and heterosis [7]. Identification of target abalone strains with higher growth rates and desirable phenotypic traits has been facilitated by molecular tracking technologies, including quantitative trait loci analysis and molecular-marker-assisted selection [8,9,10,11]. Analysis of differentially-expressed genes (DEGs) associated with the growth of marine invertebrates and molluscans led to identification of insulin-related peptide, extracellular matrix, neuropeptides, nacre protein, and immune-related proteins in *Crassostrea gigas* [12] and *Lymnaea stagnalis* [13], and immune-related genes in *Pinctada fucata* [14]. Despite these issues with abalone aquaculture, many aspects on the genetic and physiological mechanisms associated with a high growth rate in Pacific abalone remain unclear.

Recent developments in next-generation sequencing (NGS) technologies have enabled low-cost, high-throughput sequencing, which is important for research and clinical purposes [15]. High-throughput sequencing of genomes facilitates not only the discovery of genes associated with disease, but also diagnosis of pathological conditions based on rapid identification of disease-causing mutations. Recently, NGS has been used to study changes in transcriptomes according to physiological conditions or cell type in marine organisms such as zebrafish [16], salmon louse [17], and *Haliotis midae* [18]. In this study, we explored the transcriptome of Pacific abalone, *Haliotis discus hannai* siblings using NGS technology to identify the genes associated with differential growth performance. To this end, abalones of various sizes collected following growth under identical conditions were divided into large, medium, and small groups and subjected to RNAseq analysis [19]. Differentially-expressed genes were identified by comparison of the transcriptomes of the three groups and were subjected to functional annotation analysis. A proportion of the differentially-expressed genes were verified by RT-PCR.

## 2. Results and Discussion

### 2.1. Differential Growth in Pacific Abalones

Pacific abalones, *H. discus hannai*, grown for 200 days post-fertilization (dpf) under the same condition in two independent facilities, National Fisheries Research and Development Institute (NFRDI) and Marine-Fisheries and Development Institute (MFDI) as described in Materials, were subjected to transcriptome analysis. We focused our analysis on abalone spats of approximately 200 dpf, as they have distinct tissue morphologies compared to 100 dpf spats and a faster growth rate compared to 300 dpf spats, as inferred from weight increases. Therefore, 200 dpf abalones were chosen for further analysis. Eighty abalones of various sizes collected from NFRDI were measured to determine weight, shell width, length, and height, and then divide into three groups by sizes. Table 1 shows the sizes of ten abalones selected from the small-, medium-, and large-size groups subjected to RNAseq analysis. Up to a 20-fold difference in weight, together with a three-fold difference in shell length, width, and height were observed between the small- and large-size groups depending on the lineage. Another batch of Pacific abalones grown at different facility (MFDI) for 200 dpf was collected to verify the results using RT-PCR. Abalones were divided into two groups by size: small and large. Weight, shell width, length, and height ranged from 0.37 ± 0.87 g, 8.25 ± 2.02 mm, 10.98 ± 3.46 mm, and 2.63 ± 0.80 mm, respectively, in the small group (*n* = 19), to 3.79 ± 0.9 g, 22.82 ± 1.74 mm, 32.70 ± 3.41 mm, and 9.37 ± 0.94 mm, respectively, in the large group (*n* = 10). Abalones randomly selected from each group were subjected to RNA isolation followed by RT-PCR amplification. In addition, abalones older than one year with fully developed gonad phenotypes obtained from a regional market were used to examine gender-specific expression.

**Table 1:** Growth traits of Pacific abalones used for RNAseq transcriptome analysis. The sizes of 80 abalone spats 200 days post-fertilization were measured. The values (mean ± standard deviation) represent those from 10 abalones selected from small, medium, and large sized individuals for RNAseq analysis.

Groups	Shell Length (mm)	Shell Width (mm)	Shell Height (mm)	Weight (g)
Small (*n* = 10)	13.53 ± 1.34	9.54 ± 1.11	2.87 ± 0.41	0.26 ± 0.09
Medium (*n* = 10)	24.12 ± 2.49	16.35 ± 1.45	4.95 ± 0.46	1.43 ± 0.40
Large (*n* = 10)	36.15 ± 2.21	24.99 ± 1.43	8.86 ± 1.19	5.24 ± 1.09
Mean ± SD (*n* = 80)	24.63 ± 8.17	17.09 ± 5.58	5.54 ± 2.26	2.10 ± 1.81

### 2.2. Transcriptome Analysis

**Figure 1:**
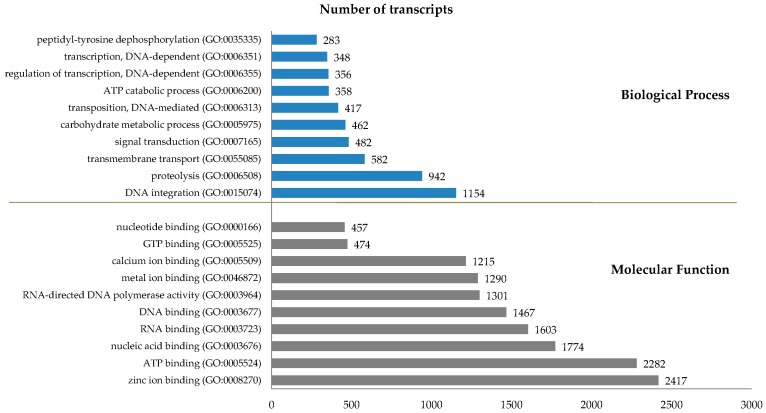
GO (Gene ontology) classification of the reference genes identified by transcriptome analysis of Pacific abalones. The top 10 GO terms and their proportions in the molecular function and biological process categories are shown.

To identify the genes associated with differential growth, RNA was isolated from soft tissues of abalone and subjected to gel electrophoresis (data not shown). RNAseq analysis was carried out using a mixture of RNAs from 10 abalones per group to reduce the problems associated with deviation stemming from a single organism. Transcript levels were quantified by calculating reads per kilobase of exon per million mapped reads (RPKM) values with a 0.3 RPKM threshold value for differential expression [20]. A total of 117,537 reference contigs (average sequence length, 804.5 bp) was obtained through *de novo* assembly together with BLASTx analysis. Of the 28,981 (24.7%) best-hit BLASTx transcripts, GO assignment revealed 17,872 (61.7%) transcripts with molecular functions (MF), the top 10 of which were (in order of magnitude): zinc ion binding (16.9%), ATP binding (16.0%), and nucleic-acid binding (12%: Figure 1). The total of 12,647 (43.6%) transcripts in the biological process (BP) category comprised predominantly those involved in DNA integration (21.4%) and proteolysis (17.5%).

Differentially-expressed transcripts were determined by comparison of the RPKM values within fold change (FC) of genes among the size groups (Table 2). The number of transcripts showing more than twofold (false discovery rate, FDR ≤ 0.05) changes in expression was 9225 to 21,655, depending on the groups included in the comparison. A total of 971 transcripts showed >10-fold differences in expression between the large- and small-size groups, of which 543 transcripts were up-regulated and 428 down-regulated in the large-size group. Gene set enrichment analysis indicated that the transcript down-regulated to the greatest extent in large-size abalones belonged to the carnitine biosynthesis category (GO:0045329). Transcripts involved in G1/S-specific transcription in the mitotic cell cycle (GO:0000083), amylopectin biosynthesis (GO:0010021), Cul5-RING ubiquitin ligase complex (GO:0031466), and regulation of nitrogen utilization (GO:0006808) showed the greatest degrees of up-regulation in the large group. Transcripts that showed consistent changes in their patterns of expression in similar comparisons; e.g., large *vs.* medium and large *vs.* small, were selected for further analysis.

**Table 2:** Genes showing differential expression between small and large abalones. Differentially-expressed transcripts were determined from comparisons of the RPKM values of genes in large *vs.* medium, large *vs.* small, and medium *vs.* small Pacific abalones. Numbers were obtained using two-, four-, and 10-fold change (FC) cut-off values, and the FDR threshold is indicated.

Comparison (Total: RPKM ≥ 0.3)		FC ≥ 2, FDR ≤ 0.05			FC ≥ 4, FDR ≤ 0.01			FC ≥ 10, FDR ≤ 0.001		
		Up	Down	Total	Up	Down	Total	Up	Down	Total
Large *vs.* Medium	22,680	4383	4842	9225	1301	908	2209	190	141	331
Large *vs.* Small	24,414	10,957	4650	15,607	2859	1545	4404	543	428	971
Medium *vs.* Small	22,338	16,406	5249	21,655	4216	1907	6123	812	514	1326

### 2.3. Reverse Transcription-PCR (RT-PCR) Analysis

RT-PCR was carried out to confirm the differences in expression detected by RNAseq analysis. The ribosomal protein L3 (Tsc1) transcript was selected from those expressed at a constant level in all three groups (0.8–1.2 FC) as an internal control for normalization of expression levels. Preliminary sets of PCR experiments were carried out with various numbers of PCR cycles to find the condition where the amounts of the products were proportional to the templates and cycles. PCR products were resolved and detected under the gel electrophoresis condition suitable for densitometric analysis. Target genes selected were those showing (i) a consistent and higher level of expression in comparisons of the large *vs.* medium, medium *vs.* small, and large *vs.* small groups; (ii) a size of >300 bp to enable design of primers that result in amplification of ≥250 bp fragments to facilitate detection; and preferably (iii) an association with genes potentially involved in growth-related phenotypes. Based on the above criteria, 34 genes showing consistent expression profile in all comparisons, preferably with available functional annotation information, were selected (Table 3). Among these 34 genes, 27 were up-regulated and seven down-regulated in the large-size group; these were designated Tsc2 to Tsc35. PCR primers corresponding to the sequences obtained from RNAseq analysis were designed using the OligoAnalyzer software (ver. 3.1; IDT Technologies, Coralville, IA, USA).

**Figure 2:**
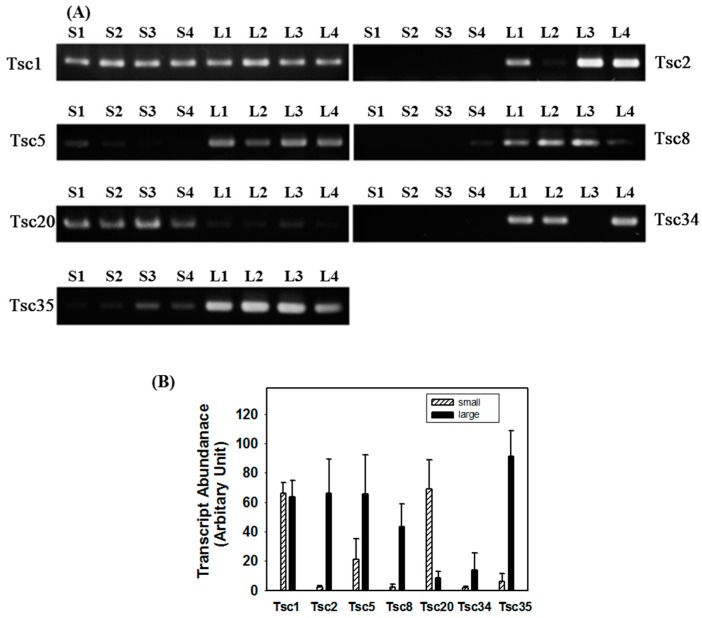
Transcript levels in small and large Pacific abalones. (**A**) RNA was isolated from four abalones in the small (S1–S4) and large (L1–L4) groups and cDNA was synthesized. PCR was carried out using primers specific for ribosomal protein L3 (Tsc1), and differentially-expressed candidates encoding transcripts similar to incilarin A (Tsc2), perlucin (Tsc5), transforming growth factor-β-induced protein ig-h3 (Tsc8), tomoregulin-2 (Tsc20), vitelline envelope zona pellucida domain 4 (Tsc34), and defensin (Tsc35). PCR products were resolved by 2% agarose gel electrophoresis followed by ethidium bromide staining; and (**B**) transcript levels in small (shaded) and large (filled) abalones. Values are expressed as the means with 95% confidence intervals (*n* ≥ 8).

Differential-expression of the selected transcripts was confirmed first using cDNA templates prepared from the RNA used for DEG analysis. To further verify the result, duplicate sets of RT-PCRs were performed using templates independently prepared from four abalones each in the small- and large-size groups from a separate batch of Pacific abalones (Figure 2). Densitometric analysis of PCR products indicated that, of the 34 transcripts (Tsc2–Tsc35) tested, six showed consistent expression profiles by both RT-PCR and RNAseq analysis. These included transcripts encoding a gene similar to incilarin A (Tsc2), perlucin (Tsc5), transforming growth factor-β-induced protein immunoglobulin-heavy chain 3 (ig-h3:Tsc8), tomoregulin-2 (Tsc20), vitelline envelope zona pellucida domain 4 (Tsc34), and defensin (Tsc35). The average (95% Confidence Interval) transcript levels calculated using at least eight individual samples from the small and large groups, respectively, were 2.30 (1.49, 3.21) and 65.95 (42.31, 89.59) for Tsc2, 21.13 (6.96, 35.40) and 65.62 (38.98, 92.26) for Tsc5, 2.19 (0.36, 4.02) and 43.35 (27.69, 59.01) for Tsc8, 68.92 (49.09, 88.78) and 8.41 (3.98, 12.84) for Tsc20, 1.65 (0.52, 2.78) and 13.77 (1.85, 25.69) for Tsc34, and 5.95 (0.48, 11.42) and 91.25 (73.59, 108.91) for Tsc35. Thus, Tsc20 was down-regulated, while the other five were up-regulated, in the large abalone group. The mean differences in the levels of all transcripts tested between the small and large groups were statistically significant (Mann-Whitney U statistic, *p* < 0.05) even with the small (*n* = 8~12) sample sizes and different variations. The absence of Tsc34 in some abalones might be due to discrepancies in its expression according to sex (see below).

**Table 3:** Genes tested for differential expression by Reverse Transcription-PCR (RT-PCR). Transcript levels identified by RNAseq analysis of large, medium, and small size abalones were shown for RPKM values. The Tsc1 housekeeping gene, which encodes ribosomal protein L3, was included as a control. Nucleotide sequences of primers used for RT-PCR were indicated. *E* value refers to the number of random hits one can expect by chance for the query sequence upon searching a database.

Gene ID	RPKM			*E* Value*	Description	bp	Primer 5′–3′
	Large	Medium	Small				
Tsc1	1294.33	1227.59	1392.61	0	Ribosomal protein L3 (D1M828)	1401	F: 5′-TGTCACCATCCTTGAGGCAC
							R: 5′-CAGGAACAGGCTTCTCCAGG
Tsc2	10.03	4.08	0.88	5.00 × 10^−16^	Incilarin A (B6RB57)	709	F: 5′-GGCGGCTACCTGGTTGAAAT
							R: 5′-CCCACGATGTCTGGTCCATC
Tsc3	0.7	0.19	0.04	2.00 × 10^−18^	Multiple epidermal growth factor-like domains 6	451	F: 5′-GTACTGCCAGCTGGGTTCAT
							R: 5′-CACCCGCCATTACAGCCAT
Tsc4	0.08	0.99	0	2.00 × 10^−23^	Multiple epidermal growth factor-like domains 11	488	F: 5′-CGGGTGGTTTGGAGAAGAGT
							R: 5′-AGGCATGATCCACAATGCGA
Tsc5	19.26	11.71	2.74	7.00 × 10^−52^	Perlucin (P82596)	375	F: 5′-CCTCTTGGGTTTATGCAGCAC
							R: 5′-CGGACTGTCTCATTTCCAGAC
Tsc6	8.59	6.11	1.86	5.00 × 10^−15^	EGF-like domain containing protein	1222	F: 5′-CACGGACATCACTGCAATAAGC
							R: 5′-GACATGGCTCATGCGTCTCA
Tsc7	4.08	1.55	0.95	2.00 × 10^−21^	Multiple epidermal growth factor-like domains 10	529	F: 5′-GCAAGCACTGCAACCAAACT
							R: 5′-TGCAACTTCTCTCCAGGCAG
Tsc8	33.68	23.4	7.37	3.00 × 10^−8^	Transforming growth factor-β-induced protein ig-h3 (K1PUI7)	399	F: 5′-GTACGCATGGTCACCTACCC
							R: 5′-TCGTAGCAGGGGTATGTTGTTA
Tsc9	2.27	1.14	0.43	4.00 × 10^−18^	EGF-like domain containing protein	745	F: 5′-CTGACTGCAAGAGAGGCTGG
							R: 5′-CTTGACATCCCCCTGGACAC
Tsc10	0.54	0.42	0.16	1.00 × 10^−38^	Insulin-like growth factor-binding protein	1123	F: 5′-AGAGGTTCCTCCTGCCATCA
							R: 5′-GCTTGTAGAGGCACCAGTGT
Tsc11	2.71	5.29	0.15	2.00 × 10^−51^	Peroxidasin-like protein	938	F: 5′-ACAGTCTCGATCTGATGGCTT
							R: 5′-CCAGAATCTGTCGCCCACTT
Tsc12	37.27	24.87	7.63	7.00 × 10^−11^	C-type lectin 4	430	F: 5′-CCTCGGGCTCTGCGACTC
							R: 5′-ACGCCAGTCACCCTCAGTTG
Tsc13	19.42	21.52	4.74	2.00 × 10^−30^	Choline transporter-like protein 1	289	F: 5′-CGTGGCAGCGATTAACTGTG
							R: 5′-GCTCATGTAGTAAGGCCGCT
Tsc14	8.14	9.34	1.86	5.00 × 10^−79^	Fibroblast growth factor receptor 2	1807	F: 5′-TCTCTGCGTCGCCATTCTTT
							R: 5′-CAGTTGGACCAGCGGAGAAT
Tsc15	2683.36	2020.81	979.78	1.00 × 10^−36^	Dermatopontin 3	604	F: 5′-TCCCTACTGCGTTTCTGCTG
							R: 5′-CTACCTTGCAGGGAGCAACA
Tsc16	49.8	17.74	16.35	3.00 × 10^−16^	Perlucin A2 protein	658	F: 5′-TCCACTGGGATTTGTGGAACA
							R: 5′-GTTGGCCCCTTCTCCAGTTG
Tsc17	36.31	46.58	9.19	2.00 × 10^−16^	CD63 antigen	1413	F: 5′-TGGATTCAGCAAACCCGGAA
							R: 5′-CAGGAAGCTTTGGGGTCGAA
Tsc18	329.2	276.28	216.62	2.00 × 10^−144^	Cathepsin L2 cysteine protease; EC	1090	F: 5′-GTCGACTGGAGAACCAAGGG
							R: 5′-GACAGAGATGGGACCAACGG
Tsc19	212.38	144.81	1636.37	0.00000007	Calcium binding protein 1	457	F: 5′-GGTGATAACCGTCTGACCGA
							R: 5′-GGTGACAAATTCGCTTCGGG
Tsc20	45.46	25.82	374.98	1.00 × 10^−23^	Tomoregulin-2 (K1Q9X6)	722	F: 5′-ACTGTGACCATGAGCGTGAG
							R: 5′-CAGCTACGTGGAGATCACGG
Tsc21	5.08	2.92	157.67	1.00 × 10^−10^	Fasciclin domain-containing protein	617	F: 5′-TCCAAGACCAGCACCCAATC
							R: 5′-GGTCTGACAGGGAACTTGGG
Tsc22	0.97	1.11	62.3	0.0000006	Fasciclin domain-containing protein	525	F: 5′-CAGGGCTCAACACCCAACAT
							R: 5′-TAGTTGATGTCGGCAGCCTT
Tsc23	1.77	1.23	12.55	2.00 × 10^−79^	Cyanophycinase	1804	F: 5′-GGAGCTCAGGAGTCGTTGAC
							R: 5′-CGTTGCGCAAGCTGTAGTAG
Tsc24	4.59	7.14	31.98	1.00 × 10^−35^	Cathepsin L	1062	F: 5′-ACTGACACGGAGGAGATCCA
							R: 5′-TCCAGTCGACTTTTGTGGGG
Tsc25	1.37	1.87	7.24	1.00 × 10^−61^	EGF-like domain-containing protein 1	2194	F: 5′-GGAGTGCAACGGAGAGAACA
							R: 5′-TCTGATGTGTTGGCAGCGAT
Tsc26	314.26	479.57	24.62	4.00 × 10^−18^	uncharacterized protein; Flags: Fragment	306	F: 5′-ATGACTGCCAGGTTTCTCCC
							R: 5′-CCCAGCAAGCATGTACAAGTG
Tsc27	255.69	183.9	24.21	1.00 × 10^−16^	Uncharacterized protein	1000	F: 5′-ACTGTCTCTACAACAGCGCC
							R: 5′-GCCTGACTCGAAGATGCAGA
Tsc28	188.03	95.65	16.94	3.00 × 10^−42^	Uncharacterized protein	611	F: 5′-ACAACTGCTCTGCTCCAACA
							R: 5′-GGTGAGTCTTCCCTTCCCAC
Tsc29	156.32	133.95	8.05	3.00 × 10^−35^	Uncharacterized protein	423	F: 5′-CAGAGCATCTGAGGGAGTGC
							R: 5′-TGCACTGACCCGAATACACC
Tsc30	14.7	15.87	2.59	0	Dual oxidase	4918	F: 5′-CACTTTGATTCCGTCGGGGA
							R: 5′-CAGCCATGGGTTGATGTCCT
Tsc31	10.29	12.05	2	4.00 × 10^−61^	Homeobox protein engrailed	1804	F: 5′-TGGGTTTTCTGCACTCGCTA
							R: 5′-TGGAACCGGTGCTCTTCTTC
Tsc32	8.17	11.22	0.83	2.00 × 10^−167^	Putative ammonium transporter 1	1512	F: 5′-GGACGATTCCACCCGGATAC
							R: 5′-AAAGGAGATGGCGGCAAAGA
Tsc33	72.4	52.73	6.98	2.00 × 10^−65^	Annulin	1013	F: 5′-GAAACTGGGGCCACGAAGTA
							R: 5′-CCATGCTTTGACTCCGACCT
Tsc34	2.28	0.6	0	0	Vitelline envelope zona pellucida domain 4 (A0MCN4)	990	F: 5′-ATCAGCTGCTACTTCCAGCC
							R: 5′-ACGGATCCCCATTCACGATG
Tsc35	155.23	89.83	33.46	1.00 × 10^−32^	Defensin (D3UAH2)	614	F: 5′-CTGCTTCTGCTGTGTTTGGT
							R: 5′-CGACAGACACAGACGCCATT

### 2.4. Tissue-Specific Expression

**Figure 3:**
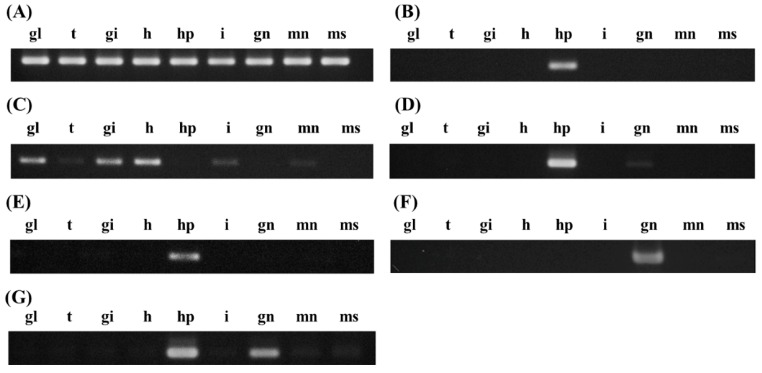
Tissue-specific distribution of transcripts whose differential expression was associated with the growth of Pacific abalone. RT-PCR was carried out using primers specific for ribosomal protein L3 (Tsc1, **A**); and differentially-expressed candidate genes encoding transcripts similar to incilarin A (Tsc2, **B**); perlucin (Tsc5, **C**); transforming growth factor-β-induced protein ig-h3 (Tsc8, **D**); tomoregulin-2 (Tsc20, **E**); vitelline envelope zona pellucida domain 4 (Tsc34, **F**); and defensin (Tsc35, **G**). Total RNAs isolated from ganglion (gl), tentacle (t), gill (gi), heart (h), hepatopancreas (hp), intestine (i), gonad (gn), mantle (mn), and muscle (ms) were used for cDNA synthesis, followed by PCR amplification.

**Figure 4:**
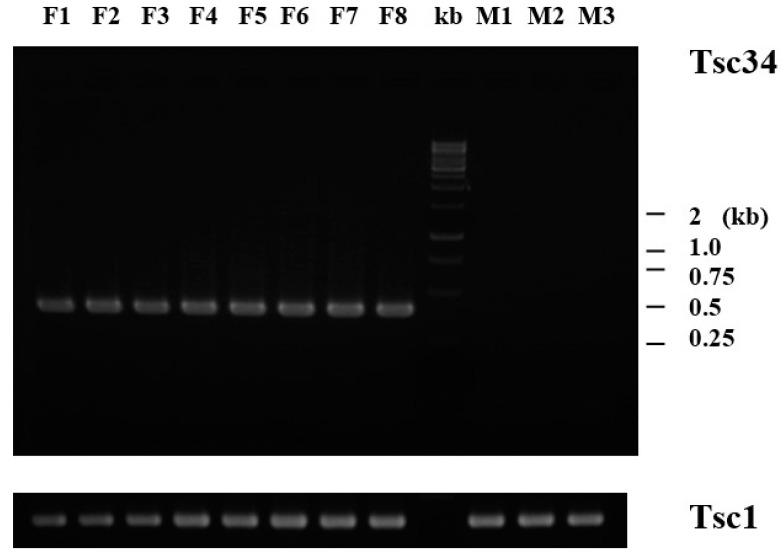
Female-specific expression of Tsc34, which encodes vitelline envelope zona pellucida domain 4. Total RNAs were isolated from gonads of fully grown abalones that exhibited eight female (F1–F8) and three male (M1–M3) gonads. RT-PCR was carried out using cDNA templates and primers specific for ribosomal protein L3 (Tsc1), and vitelline envelope zona pellucida domain 4 (Tsc34). Sizes of molecular weight (kb) markers are indicated.

To examine tissue-specific expression profiles, the ganglion, tentacle, gill, heart, hepatopancreas, intestine, gonad, mantle, and muscle were dissected from adult *H. discus hannai.* RNA isolated from each tissue was used for cDNA synthesis using oligo-dT primers, as described above (Figure 3). While the Tsc2, Tsc8, and Tsc20 transcripts were expressed mainly in the hepatopancreas, Tsc35 was expressed at high levels in the hepatopancreas and gonad. Tsc5 was expressed mainly in the ganglion, gill, and heart and at a low level in the tentacle, intestine, and mantle. The predominant expression in the hepatopancreas suggests involvement of this organ in production or secretion of growth-related peptides and induction of somatic growth by stimulating various tissues. In particular, Tsc34 expression was detected only in gonads (Figure 3F) and differed according to its gender (Figure 4, see below). The latter finding is consistent with a previous report of ZP4 expression in growing oocytes [21,22]. To verify female-specific expression, gonads were isolated from sexually matured abalones, which were readily identifiable by eye and microscopy. RT-PCR amplification of cDNA templates prepared from at least three male and eight female abalones indicated that Tsc34 was expressed in female, but not in male, gonads (Figure 4). This female-specific expression profile of Tsc34 may explain the absence of its expression in some large male abalones (L3 in Figure 2), as no distinction between sexes was made, which resulted in a mixed population analyzed in the experiment. Absence of Tsc34 in small abalones might result from an immature development of gonads in a small size group of 200 dpf abalones.

### 2.5. Functional Implications

Tsc2 showed 11.4-fold (RNAseq) to 30-fold (RT-PCR) higher expression in the large compared to the small groups (Table 3). Sequence analysis of Tsc2 indicated similarity to incilarin A (UniProt ID: B6RB57) and C-type lectin (CTLs) (UniProt ID: G8XSQ0). Carbohydrate recognition domains in CTLs facilitate pathogen recognition by specifically binding to cell-wall carbohydrates in a Ca^2+^-dependent manner [23]. Recombinant CTLs cloned from the family Haliotidae showed a high binding affinity to Gram-negative bacterial pathogens [24,25]. Invertebrates rely mainly on innate immunity as they lack a complete adaptive immune system; therefore, CTLs in molluscans play important roles in recognition of invading pathogens [26]. CTLs from shrimp were reported to be expressed mostly in the hepatopancreas following bacterial challenge, suggesting that pathogen infection triggers production of hepatopancreas-specific CTLs for host defense [27,28,29,30,31,32]. A recent study of pearl oyster demonstrated a directly proportional relationship between lectin gene expression and oyster growth using RNAseq and qRT-PCR [14]. Therefore, Tsc2 may contribute to abalone growth by a mechanism similar to that of lectin and may also play a role in Ca-mediated cellular processes.

Tsc5 expression was 4–7-fold (RNAseq analysis) and 3-fold (RT-PCR) higher in the larger size group depending on the analysis. The sequence of Tsc5 showed marked similarity to that of *Haliotis laevigata* perlucin (UniProt ID: P82596), which plays a vital role in shell formation by accelerating CaCO_3_ precipitation and biomineralization [33]. Perlucin contains a Gln-Pro-Asp (QPD) sequence, *i.e.*, a conserved carbohydrate-binding motif, in the carbohydrate recognition domain [34,35]. Tsc5 was expressed mainly in the ganglion, gill, and heart, and at a low level in the tentacle, intestine, and mantle. Enzymes involved in calcium-mediated biomineralization processes have been reported to be expressed at high levels in the gill, mantle, and hemolymph of *Pinctada margaritifera* [36]. In the freshwater pearl mussel *Hyriopsis cumingi*, perlucin was expressed primarily in the mantle, adductor, gill, and hemocytes, which are the main compartments participating in calcium metabolism and shell formation [37]. This result is consistent with the notion that growth is strongly dependent on the levels of calcium ions, as these are important for shell formation in mollusks. Therefore, Tsc5 may play a vital role in abalone growth by accelerating the calcium-dependent processes involved in shell formation and growth.

The expression of Tsc8, which encodes a transcript similar to the transforming growth factor-beta-induced protein (TGFBIP) ig-h3, was up to 4.6-fold (RNAseq analysis) in large abalones. Similar tendencies of up-regulation (~20-fold) was also shown in RT-PCR analysis. TGFBIP is an extracellular matrix protein that binds to collagens and is involved in cell adhesion and endochondrial bone formation in cartilage to cancers. It is also an extracellular component of developing ligaments, aorta, lung and kidney, mature cornea, skin, bladder, blood, and bone of animals. *TGFBIp* expression was detected primarily in the corneal epithelial cells. The association of Tsc8 with the stroma during development and wound healing suggests its possible involvement in animal growth.

Tsc20 encodes a gene similar to that encoding tomoregulin-2, a transmembrane protein expressed predominantly in the brain of mammals. Expression analysis showed up to 14.5-fold (RNAseq) and 8.2-fold (RT-PCR) down regulation of Tsc20 in large abalone. The presence of modules involved in binding to TGF-β-related growth factor, an EGF-like domain, and a cytoplasmic domain containing a potential G-protein–activating motif in tomoregulin suggest its involvement in cellular signaling cascades that lead to growth promotion. The protein appears to promote survival of hippocampal and mesencephalic neurons in primary culture [38]. It has also been suggested as being involved in tissue growth and repair in the central nervous system, and its expression was up-regulated under conditions that promoted the reinitiation of primary dendrites on mature cortical neurons [39] during androgen-independent growth of prostate cancer [40]. These findings suggest a role for Tsc20 in the growth of abalone.

Tsc34 encodes a gene similar to vitelline envelope zona pellucida domain 4 of the California red abalone (*Haliotis rufescens*) (UniProt ID: A0MCN4), which is a major component of the extracellular coating of abalone eggs [41]. Our RNAseq analysis showed marked Tsc34 expression in large-size abalones, whereas no expression was detected in small-size abalones. This expression pattern was confirmed by the RT-PCR results. The proportion of Tsc34-expressing individuals was higher in the large-size group than the small-size group, and it was found to be expressed specifically in females. Therefore, Tsc34 may represent not only a useful marker for evaluation of abalone growth rates and sex determination but also enables determination of differences in growth rates between male and female abalones.

The sequence of Tsc35 showed similarity to that of defensin (UniProt ID: D3UAH2), and harbored six conserved cysteine domains [42,43]. The expression of Tsc35 was up-regulated ranging from 4.6-fold to 15-fold in RNAseq and RT-PCR analyses, respectively, in large-size abalones. The innate immune system of invertebrates is vital for defense against pathogen invasion because of their lack of adaptive immunity. Defensins consisting of 3–6 kDa cationic peptides are classified as small antimicrobial proteins (AMPs), and are important for host defense [44,45]. Defensin was expressed in the hepatopancreas and gonads; its expression in the hepatopancreas is similar to a previous report [46]. Therefore, Tsc35 together with Tsc2 may increase the growth rate of abalones by enhancing their immunity.

## 3. Experimental Section

### 3.1. Materials

Pacific abalones, *H. discus hannai*, were collected from the Genetics and Breeding Research Center of the National Fisheries Research and Development Institute (NFRDI, Geoje, Korea) and from the Abalone Research Institute of Jeolla Namdo Marine-Fisheries and Development Institute (MFDI, Wando, Korea). Rearing of abalones was carried out as described [47]. For gender-specific experiments, abalones older than one year with fully sexually matured gonads were obtained from a regional NamChun Mart (Busan, Korea). Total RNA isolation kits and reagents, including RiboEX™ and Hybrid-R™ columns, were obtained from GeneAll (Seoul, Korea). RNASeq and transcriptome analyses were carried out by Insilicogen Inc. (Suwon, Korea). RNase-free DNaseI was obtained from Roche (Indianapolis, IN, USA). 5XHiQ-PCR reaction mixture and oligonucleotide primers were obtained from GenoTech Corp (Daejeon, Korea), and RNase inhibitor and Moloney Murine Leukemia Virus Reverse Transcriptase (M-MLV) cDNA synthesis kits were obtained from Enzynomics (Daejeon, Korea).

### 3.2. RNA Isolation

Pacific abalones *H. discus hannai* were grown to 200 dpf under the facilities at NFRDI and MFDI as described above. Total of 80 abalones were collected from the batch of abalone grown under the same growth condition and, upon size measurements, divided into three groups according to size. Soft tissues were isolated from representative abalones randomly selected from each group and immediately frozen in liquid nitrogen and stored at −80 °C. For RNA isolation, soft tissue (~0.1 g) from each organism was homogenized in 1 mL of RiboEX™ (GeneAll) in a glass homogenizer vessel. Upon addition of 200 μL chloroform followed by centrifugation at 12,000× *g* at 4 °C for 5 min, 500 μL of the aqueous phase was mixed with the same volume of isopropanol. Following centrifugation at 12,000× *g* for 5 min, the pellet was washed with 75% ethanol, dried, and then treated with RNase-free DNase I for 20 min at 37 °C [48]. RNA was purified using a Hybrid-R™ column (GeneAll) according to the protocol provided by the manufacturer. RNA quality was assessed by agarose gel electrophoresis and a Micro-volume Nucleic Acid Spectrophotometer (ASP-2680, ver. 4.1, ACTGene Inc., Piscataway, NJ, USA).

### 3.3. Transcriptome Library Construction and Analysis of Differentially-Expressed Genes

RNAseq analysis was carried out according to the manufacturer’s protocol (Insilicogen Inc.). The procedure involves poly A^+^ RNA isolation using the Truseq RNA Sample Prep Kit (Illumina, San Diego, CA, USA), cDNA synthesis using random primers, Truseq adaptor addition to phosphorylated cDNA, and sequencing analysis by Illumina HiSeq 2000 (Illumina, San Diego, CA, USA). *De novo* transcript assembly of raw sequences was performed using the CLC Assembly Cell Package (ver. 4.2.0; CLCBio, Arhaus, Denmark) and CLC_mapper (CLC Assembly Cell). Sequence annotation and mapping were conducted using BLASTx based on the information in the UniProt *Metazoa* database with a threshold E-value of 1.00 × 10^−5^, followed by functional annotation of reference contigs and differentially-expressed genes using the Gene Ontology and Kyoto Encyclopedia of Genes and Genomes databases. Transcript levels were quantified using the reads per kilobase per million reads (RPKM) method [19], with a threshold criterion for differential expression of >0.3 RPKM [16]. Fold changes in expression and *p*-values (Audic and Clavarie’s method) were calculated by comparison of RPKM values. Based on the false-discovery rate (FDR) value obtained using the Benjamini-Hochberg method [49], differentially expressed genes with fold-change values >2 (FDR ≤ 0.05), >4 (FDR ≤ 0.01), and >10 (FDR ≤ 0.001) were enumerated.

### 3.4. RT-PCR

RNA was extracted from soft tissues of abalones (*n* ≥ 8) using TRI Reagent (Sigma, St. Louis, MO, USA) followed by the Hybrid-R™ Kit (GeneAll), as described above. First-strand cDNA was synthesized in a 20 μL reaction containing 1 μg total RNA and 0.5 μM dT_15_ using a 1 × M-MLV cDNA synthesis kit, and 20 units of RNase Inhibitor (Enzynomics). PCR was carried out in 20 μL reactions containing cDNA templates and 1 μM target-specific primers in HiQ-PCR Mix (GenoTech Corp.). PCR conditions consisted of an initial denaturation at 95 °C for 3 min, followed by 30 cycles of denaturation at 95 °C for 30 s, annealing for 30 s at the optimum temperature for each primer pair, and extension at 72 °C for 30 s (Bio-Rad Thermal Cycler T1000, Hercules, CA, USA), and a final extension for 5 min at 72 °C. As an internal positive control, ribosomal protein L3 was amplified in 28 cycles consisting of denaturation at 95 °C for 30 s, annealing at 55 °C for 30 s, and extension at 72 °C for 30 s. To evaluate the tissue-specific expression of the selected genes, the ganglion, tentacle, gill, heart, hepatopancreas, intestine, gonad, mantle, and muscle were dissected from adult *Haliotis discus hannai*. RNA isolated was used for cDNA synthesis using oligo-dT primers followed by RT-PCR, as described above.

### 3.5. Transcript Analysis and Statistics

PCR products were resolved by 2% agarose gel electrophoresis followed by staining with ethidium bromide. Amplified products were quantified using a Gel Doc System/Station (Bio-Rad) with background subtraction. Data are shown as means ± SD. Mann-Whitney U statistic was used to compare differences between the small and large groups using a probability of *p* < 0.05 as the criterion for statistical significance. Data analysis including Mann-Whitney statistic was carried out by using Statistical Package for the Social Science (SPSS) software (ver. 21; IBM SPSS Inc., Armonk, NY, USA) and SigmaPlot (Systat Software, SanJose, CA, USA). The confidence interval was calculated based on sample size, sample observed mean, and standard deviation.

## 4. Conclusions

A transcriptome analysis of the Pacific abalone, *H. discus hannai*, was performed with the aim of identifying genes associated with high growth rates, followed by molecular function, and biological process analyses. Based on transcriptome and RT-PCR analyses, a total of six differentially-expressed genes associated with high growth rates was identified in this study. The genes encoding incilarin A, perlucin, transforming growth factor-beta-induced protein ig-h3, tomoregulin-2, vitelline envelope zona pellucida domain 4, and defensin were up-regulated, while that encoding tomoregulin was down-regulated, in abalones of large size. This study provides information that will increase our understanding of the mechanisms associated with the growth of Pacific abalone and will facilitate development of molecular markers for a high-growth-rate Pacific abalone strain for the aquaculture industry.
